# Interplay of Environmental Regulation and Local Protectionism in China

**DOI:** 10.3390/ijerph19106351

**Published:** 2022-05-23

**Authors:** Guanghui Tian, Jianming Miao, Changhong Miao, Yehua Dennis Wei, Dongyang Yang

**Affiliations:** 1 College of Urban and Environmental Sciences, Xuchang University, Xuchang 461000, China; tianguanghuixcxy@163.com; 2 School of Economics, Henan University, Kaifeng 475004, China; 3 Key Research Institute of Yellow River Civilization and Sustainable Development & Collaborative Innovation Center on Yellow River Civilization Jointly Built by Henan Province and Ministry of Education, Henan University, Kaifeng 475004, China; chhmiao@henu.edu.cn (C.M.); yangdy@henu.edu.cn (D.Y.); 4 College of Geography and Environmental Science, Henan University, Kaifeng 475004, China; 5 Department of Geography, University of Utah, Salt Lake City, UT 84112-9155, USA; wei@geog.utah.edu

**Keywords:** environmental regulation, local protectionism, local competition, space panel simultaneous equation, China

## Abstract

Environmental regulation (ER) and local protectionism (LP) are important policy tools for Chinese local governments to improve the environment and promote growth, respectively, but we know little about their interplay in dealing with pollution-intensive industries and enterprises. Using spatial correlation analysis and spatial panel simultaneous equations models, we investigated the spatial characteristics and interactions of the ER and LP in China’s 285 prefectural cities. We found that the high-ER-intensity areas were spreading from the eastern to the central and western regions, and the patterns of LP transited from high in the north and low in the south to high in the west and low in the east. There was a negative correlation spatially between ER and LP. LP could inhibit the increase in ER intensity, while the continuously increasing ER intensity could restrict LP through the competitive behavior from the “race to the bottom” to the “race to the top” among local governments. The effect of ER restricting LP was significant from 2008 to 2013 and prominent in the east, which was dominated by “race to the top” competition, while LP had a greater inhibitory effect on ER in the central and western regions, which preferred to obtain tax revenues from pollution-intensive industries. The results imply that removing the roots of local protectionism, improving the environmental governance system, and formulating differentiated regional environmental regulatory measures will help local governments balance economic growth and environmental protection.

## 1. Introduction

With the rapid industrialization and urbanization, the question of how to balance economic growth and environmental protection has become an important agenda for the Chinese government. China has gradually improved its environmental management system by forming a system of coordinated pollution control by multiple regulatory bodies and regulatory tools [1,2], which has achieved some promising results. However, severe environmental violations are also observed, indicating that environment regulation (ER) is not well implemented, and the problem of low regulatory efficiency still exists [3,4,5,6]. China’s environmental governance still faces major contradictions and challenges [7,8]. 

Some scholars have tackled the environmental and regulatory problems from economic perspectives, such as the industrial scale, the industrial structure, technological innovation, agglomeration externalities, and globalization [9,10]. However, such economic investigations often de-emphasize the impact of China’s transitional background and government-led development model on the ER efficiency. More attention has to be paid to the conflict and trade-off relationship between economic development and environmental management in local government behavior [11,12,13]. Chinese local governments include provincial, municipal, county, and township levels and take the responsibility to develop the economy and protect the environment not only under their administrative rights and financial power but also under the supervision and encouragement of the central government. On the one hand, the demand for local governments to pursue economic growth is the root of LP [14]. Relaxing the ER as a local development strategy is conducive to reducing the compliance costs of polluting companies and achieving economic growth and taxation objectives. Imitating behaviors among local governments may result in a “race to the bottom” of environmental regulatory policies, which may make regional economic growth and environmental protection as a whole fall into a prisoner’s dilemma [15,16]. On the other hand, the deterioration of the environment may force local governments to raise the ER intensity with the central government’s strengthening of local vertical management and horizontal supervision [17,18]. The governance model that integrates pollution monitoring, governance, supervision, and penalties helps to break the local government’s incentivized mechanism of the protection of polluting enterprises [11], gradually eliminate the “protective umbrella” of environmental violations, and promote the phenomenon of the “race to the top” [4,19]. Therefore, analyzing the relationship between LP and ER, which has received less attention, is of great significance to understanding the conflicting goals and behavior dilemmas of local governments in pursuing economic development and strengthening environmental protection.

This paper explores the interplay between the ER and LP of local governments in China based on the conflict and interaction of local governments in economic development and environmental governance. We analyze the conflict of local government behaviors under sustainable development and regional differences [20] and identify the evolution of and problems with the ER behavior of local governments in China. This paper will also fill the void created by the lack of analysis of mutual competition strategies of local governments in prefecture-level cites [13].

## 2. Literature Review and Analytical Framework

Local protectionism refers to the policies and practices of local governments that protect local firms against competition from non-local firms for their economic growth. China’s economic reform of fiscal decentralization provided local governments with a strong incentive to protect their tax base by shielding local firms and industries from inter-regional competition and to protect state-owned enterprises under their administration [14]. Local protectionism arises from a combination of factors, including the promotion of local economies, the top-down political personnel system that relies heavily on local GDP growth for promotion, and the rent-seeking behavior of local officials [21,22]. Locally modified ER is an effective instrument for such a purpose [23], which refers to the imposition of limitations or responsibilities on individuals, corporations, and other entities to prevent environmental damage or improve degraded environments [24]. If local governments desire to develop the economy, the actual implementation of environmental policies will be greatly reduced, especially when the main tax base is comprised of pollution-intensive industries. To better address the interactive relationship between ER and LP, we sort out the literature from the following three perspectives.

### 2.1. The Interplay between ER and LP: Game between the Central and Local Governments

The principal-agent theory focuses on the autonomy and initiative of local governments to analyze the relationship between the central and local governments in China. In the context of decentralized governance, the central government decomposes and assigns environmental goals to local governments, and the efficiency of environmental governance depends on the local government’s enforcement and implementation of policies [4,12].

Due to the mismatch between fiscal and administrative power, local governments face greater financial pressure, and some local municipalities may use low-intensity ER to attract capital inflows [11,25]. Due to the information asymmetry, local governments have incentives to hide pollution information and passively implement delegated environmental policies. In particular, local governments largely control the decision-making and resource allocation for economic development, and the lack of independent local environmental protection agencies allows LP motives to affect ER policies and the implementation intensity [26,27,28].

The “tournament competition” model of government officials’ promotion and the “meeting the standard” model of environmental governance contribute to this phenomenon [23,29]. Under flexible tenure rules, local officials cannot form a joint expectation for their tenure, making them inclined to invest in economic public goods while ignoring the supply of social public goods [22]. Environmental protection and renewable energy vehicles are the two areas in which the Chinese media discloses the most LP behaviors. Only when the central government designs a feasible contractual relationship, and the dominant strategy of the game between the central and local governments evolves into mandatory intervention by the central government and local cooperation, can the unification of interests between political centralization and economic decentralization be guaranteed, encouraging local governments to attach importance to environmental goals and strictly implement an environmental policy [18,30].

### 2.2. The Interplay between ER and LP: Competition among Local Governments

The purpose of the competition among regional ER strategies is to obtain mobile production factors, such as capital, labor, and industry, and subsequently gain a competitive advantage. The loose environmental policies adopted by regions will increase local corporate profits, outputs, and welfare levels but reduce them in neighboring regions [13]. Similar behaviors in neighboring regions will lead to a “race to the bottom” competition in ER among local governments, which can cause a series of economic and environmental problems [15,31].

For example, some regions intentionally deploy polluting industries at administrative boundaries and downstream of river basins to transfer negative environmental externalities and reduce environmental regulatory costs [32,33]. However, when the pollution damage is greater than the economic welfare, the jurisdiction focuses on the use of a “relative performance” comparison to solve the information asymmetry problem, and local governments’ pursuit of “mobile factors that prefer high-quality environments” will promote the formation of a “race to the top” competition in ER [34].

The positive spillover effect of this ER and the increased awareness of cooperation under regional joint prevention and control measures will help weaken the LP of polluting enterprises and form healthy competition. At the same time, due to temporal and spatial heterogeneity, the competition behavior of ER faces the coexistence of a “race to the bottom” and a “race to the top” [19].

### 2.3. The Interplay between ER and LP: Relations between Local Governments and Firms

The interaction between firms and local governments in China is an important viewpoint of local corporatism [35]. Under the influence of fiscal decentralization, the demands of local governments for fiscal income, taxation, employment, and economic growth have prompted local governments and enterprises to form a community of interests with an “asylum relationship” [34,36].

Based on local economic and social interests and personal interests, local governments and officials use administrative power to perform “rent-seeking” and “rent-setting” in order to reduce the impact of ER on local enterprises. For example, the government can maintain the layout of polluting enterprises in the region by means of subsidies, tax relief, cheap land prices, and low-interest loans [37]. Enterprises also rely on local governments to avoid expensive investments in environmental protection and obtain advantageous resources for development. Polluting enterprises will spend a lot of energy and time dealing with their relationship with the government [38]. Meanwhile, industrial enterprises have greater bargaining power with the local government in areas where local economies are less developed [39,40].

However, the “asylum relationship” between the government and enterprises will continue to disintegrate with the improvement of the environmental legal system and the increase in anti-corruption efforts [41]. The transformation to a service-oriented and regulatory-oriented government, and the inclusion of ecological and environmental performance in the official assessment system, will strengthen the supervision of enterprises [11]. Simultaneously, enterprises settled in industrial clusters can bring economic benefits and make full use of centralized treatments of pollutants to achieve scale effects in pollution abatement and reduce pollution control costs [10,13]. Enterprises will also reduce non-productive expenditures and focus on production and environmental governance [8].

### 2.4. Analytical Framework

There is a two-way interactive relationship between LP and ER, and this relationship changes with the improvement of the government’s governance ability. However, existing theories and empirical studies are mostly based on economic models, such as game theory [18], strategic interaction models of ER [13,23], and the impact of local competition on ER [42]. Few scholars have empirically tested the interactive relationship between LP and ER.

**Figure 1:**
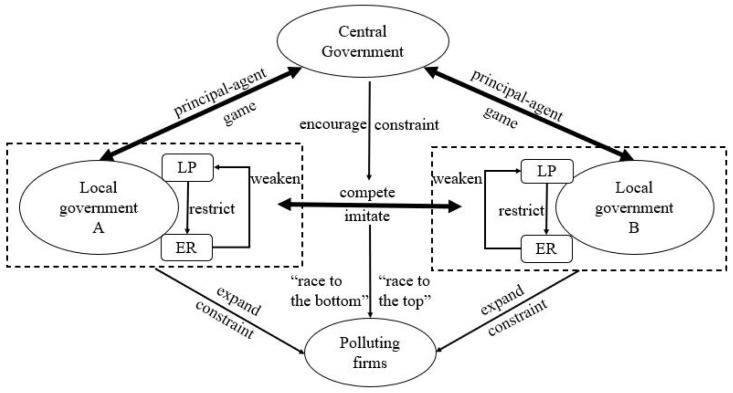
The interaction mechanisms between LP and ER.

In order to understand the interaction mechanisms between the LP and ER of Chinese local governments, we constructed an analytical framework that integrates the three actors of the central government, the local government, and the polluting firm and their complex game, competition, and asylum relations (Figure 1). In China, local government behavior relies on the administrative and economic decentralization of the central government. The official promotion using GDP as the core standard and the local financial pressure after the reform of the tax sharing system urge the local government to adopt economic growth and tax growth as the core goals. Due to the externality of environmental pollution, local governments prefer to protect local polluting enterprises to promote economic growth and do not pay attention to environmental protection. In order to compete for key production factors, local governments will perform similar actions to implement local protectionism and reduce the level of environmental regulation. Therefore, we believe that the behavior of LP based on local economic growth and fiscal and tax goals has a negative effect on ER, which could lead to “race to the bottom” competition.

However, with the increasingly serious environmental problems, the central government has increasingly emphasized the win–win goal to achieve environmental protection and economic growth ever since the 17th National Congress of the Communist Party of China in 2007. The weight of the environmental outcomes in the assessment system of the central government for local leaders is increasing. At the same time, the central government has strengthened the construction of the environmental governance system and further trusted local governments to strictly fulfill their environmental protection responsibilities. This will not only change the competition basis of local governments and strengthen ER, but also restrict the behavior of local polluting enterprises, as local governments will compete to improve the level of ER. Therefore, we hold that with increasingly strict environmental governance, ER will have a negative effect on LP, which can cause “race to the top” competition.

In addition, there are great differences in the level of economic development and the environmental governance ability in various regions of China, and the competitive behaviors of local governments are also different. Therefore, we propose that there exist heterogeneities in time and space in the interaction mechanisms between ER and LP. In the developed eastern region, the enhancement of local governments’ awareness of environmental protection will weaken the motivation to protect polluting enterprises and the competitive behavior of local governments will be more inclined to “race to the top”, while in the underdeveloped central and western regions, the stronger motivation to protect local polluting enterprises will restrict the improvement of the environmental regulation level, and the competitive behavior of local governments will be more inclined to “race to the bottom”.

## 3. Study Design

### 3.1. Measurement of ER Intensity

Due to the differences in research perspectives and scales, there are multiple nonunified quantitative methods for measuring ER, including the comprehensive index [23], the substitution method [10], and the natural difference method [39]. Considering that multidimensionality and comparability are two relatively important aspects of the measurement of ER, we constructed an ER intensity index based on Keller’s industrial composition pollution abatement adjusted cost measurement approach [43]. The main goal is to measure the effectiveness of local government ER by comparing the predicted pollution emission intensity based on two-digits industries and the real pollution emission intensity. We assumed that the emission intensity of regional two-digits industries is consistent with that of national industries and that the changes in emission intensity caused by industrial green technological innovation and corporate green transformation are attributed to the pollution control efforts of local governments. The steps are as follows:

First, we calculate the actual emission intensity *S_jst_*:

(1) $$S_{sjt}=\frac{P_{sjt}}{Y_{st}}$$

where *P_sjt_* refers to the emissions of pollutant *j* of city *s* in year *t*, and *Y_st_* refers to the industrial output of city *s* in year *t*.

Secondly, we use the output value of 40 industries in prefecture-level cities and the country’s overall industrial emission intensity to obtain the pollution emission intensity predicted for city s:

(2) $${\overset{⌢}{S}}_{\mathrm{sjt}}=\frac{1}{Y_{st}}\sum_{i=1}^{40}\frac{Y_{sit}P_{ijt}}{Y_{it}}$$

where *Y_sit_* is the output of industry *i* of city *s* in year *t*, *P_ijt_* refers to the emissions of pollutant *j* of industry *i* in year *t*, and *Y_it_* is the total industrial output value of industry *i* in year *t* for the whole nation.

Finally, we compare the predicted pollution emission intensity with the actual pollution emission intensity of the city, and incorporate three pollutants (industrial wastewater, industrial sulfur dioxide, and industrial smoke (dust)) to construct a comprehensive index of the city’s ER intensity:

(3) $${ER}_{sjt}=\frac{{\overset{⌢}{S}}_{\mathrm{sjt}}}{S_{sjt}},\;ER_{st}=\frac{1}{3}(ER_{stj1}+ER_{stj2}+ER_{stj3})$$

where *ER_sjt_* is the pollution control efforts for pollutant *j* in city *s* in year *t,* and j includes industrial wastewater, industrial sulfur dioxide, and industrial smoke (dust). *ER_st_* is the comprehensive index of ER intensity for city *s* in year *t*. The higher the *ER* value, the stronger the government’s environmental governance.

### 3.2. Measurement of the LP Index

LP currently has no unified measurement method. There are no complete statistics on the flow of trade, economy, and factors between two cities, and LP is increasingly inclined to adapt using covert means. In the literature, scholars have constructed the indicators of the degree of regional or industrial LP from a certain perspective to tackle specific research problems, which can be summarized into the following methods: methods based on the industrial structure and production efficiency [44]; methods based on consideration of LP motivations [14]; methods based on commodity and trade flow measurements; methods based on the relative price difference [45]; and methods based on a questionnaire survey.

The primary objective of this study is to investigate the interactive relationship between LP and ER, so we used the motivation of local governments to protect pollution-intensive industries to construct the LP index. Pollution-intensive industries are the main targets of ER and an important driver of local economic growth, taxation, and employment [3]. From the perspective of the mismatch between local fiscal power and administrative power, it is considered that local governments have incentives to support, subsidize, or protect polluting industries with high tax revenue in their jurisdictions. This may hinder the implementation of ER policies. Thus, we used the proportion of city-level total tax revenue from pollution-intensive industries to the regional fiscal revenue to measure the degree of protection of pollution-intensive industries by local governments. The formula is:

(4) $${DF}_{it}=\frac{{YE}_{it}+{ZZ}_{it}*25\%+{SD}_{it}*40\%}{{FR}_{it}}$$

where *i* and *t* represent the city and year, respectively; *YE_it_*, *ZZ_it_*, and *SD_it_* are the main business taxes and surcharges, the value-added tax, and the corporate income tax, respectively; and *FR_it_* represents the local fiscal revenue. For a long period of time, local governments receive 25% of the value-added tax paid by firms and 40% of the corporate income tax. The main business taxes and surcharges are mostly local taxes. Therefore, we adjusted the value-added tax and the corporate income tax to reflect the impact of local taxes on the degree of local government dependence on pollution-intensive industries.

In this study, the selection of pollution-intensive industries was performed according to the pollution intensity index (*EPI*) of three pollutants (industrial wastewater, industrial waste gas, and industrial solid waste) in the industries in China from 2003 to 2013.

*EPI* = (*E* × *P*)^1/2^ (5)

where *E* is the pollution emission intensity (pollutant emissions per unit industrial output value) and *P* is the industrial scale (the proportion of pollutant emissions of an industry in the total pollutant emissions of the country). Finally, the pollution-intensive industries used in this study include agricultural and sideline food processing, paper and paper products, textiles, beverage manufacturing, petroleum processing, coking and nuclear fuel processing, chemical raw material and chemical product manufacturing, chemical fiber manufacturing, non-metallic mineral manufacturing, ferrous metal smelting and rolling processing, non-ferrous metal smelting and rolling processing, electric power, and heat production and supply, which are relatively consistent with the first nationwide pollution source survey (2007) launched by China’s State Council [4].

### 3.3. Bivariate Spatial Autocorrelation

Bivariate spatial autocorrelation has a great advantage in describing the spatial correlation pattern and dependence characteristics of ER and LP. If there is a spatial autocorrelation between ER and LP, we can explore the impact of ER spatial lag (or LP spatial lag) on LP (or ER) in spatial simultaneous equations. There are two types of Moran’s *I* statistics: global and local. Bivariate global autocorrelation is used to identify the spatial relationship between two variables in the whole research area [46]. The expression is:

(6) $$I_{ap}=\frac{n\sum_{i=1}^{n}\sum_{j\neq i}^{n}W_{ij}Z_{i}^{a}Z_{j}^{p}}{(n-1)\sum_{i=1}^{n}\sum_{j\neq i}^{n}W_{ij}}$$

where *n* is the number of samples and *W_ij_* is the spatial weight matrix constructed based on the proximity criterion. The queen contiguity spatial weight matrix was adopted. $Z_{i}^{a}$ and $Z_{J}^{P}$ represent the normalized data of the mean value of the observed value of attribute *a* of the city *i* and the normalized value of the observed value of attribute *p* of the area *j* adjacent to *i*, respectively. The value range of *I_ap_* is between −1 and 1. If *I_ap_* > 0, it means that the geographic elements *a* and *p* have a positive spatial relationship, and if *I_ap_* < 0, it means that the two have a negative spatial relationship.

Bivariate local spatial autocorrelation analysis can identify the spatial correlation pattern of two variables in a local area [47]. Its expression is:

(7) $$I_{i}^{ap}=Z_{i}^{a}\sum W_{ij}Z_{j}^{p}$$

where $I_{i}^{ap}$ depicts the relationship between city *i*’s geographic attribute *a* and peripheral region *j*’s weighted average attribute *p*. If $I_{i}^{ap}$ is significantly positive, it indicates that *a* and *p* have a positive correlation; if the $I_{i}^{ap}$ value is significantly negative, then it is a negative correlation; and if the result is not significant, it indicates that there was no obvious correlation. $I_{i}^{ap}$ can be further divided into High–High (HH), Low–Low (LL), High–Low (HL), and Low–High (LH) aggregation types. It should be noted that bivariate autocorrelation analysis needs to specify lag variables. When analyzing the bivariate autocorrelation, we calculated the global autocorrelation and local autocorrelation results of ER lag and LP lag, respectively.

### 3.4. Spatial Panel Simultaneous Equation Model

In order to verify the relationship between LP and ER and explore the local competition strategies and feedback from neighbors, we used ER and LP as endogenous variables to construct a spatial simultaneous equations (SSEs) model. The SSEs model not only considers the spatial autocorrelation of endogenous variables, but also takes the feedback and spatial spillover between endogenous variables into account [47]. It can also be used to avoid the bias that results from single-equation regression due to the endogeneity issue. The formula is as follows:

(8) $$\begin{array}{l} {ER}_{\mathrm{it}}=\alpha_{0}+\alpha_{1}\sum_{j\neq i}^{n}W_{ij}{ER}_{it}+\alpha_{2}\sum_{j\neq i}^{n}W_{ij}{Gov}_{it}+\alpha_{3}{Gov}_{it}+\alpha_{4}X_{1it}+u_{1i}+\lambda_{1t}+\varepsilon_{1it}\\ {Gov}_{it}=\beta_{0}+\beta_{1}\sum_{j\neq i}^{n}W_{ij}{Gov}_{it}+\beta_{2}\sum_{j\neq i}^{n}W_{ij}{ER}_{it}+\beta_{3}{ER}_{it}+\beta_{4}X_{2it}+u_{2i}+\lambda_{2t}+\varepsilon_{2it} \end{array}$$

In Equation (8), *ER_it_* and *Gov_it_* represent the ER intensity and the LP index in year *t* of city *i*, respectively; *n* represents the number of cities; *X*_1_ and *X*_2_ are exogenous control variables; *α*_0_ and *β*_0_ are constant terms; *μ* and *λ* are the individual fixed effect and the time fixed effect, respectively; and *ε* is a random disturbance term. In the model, *α*_1_ and *β*_1_ can reveal the spatial spillover effects of ER and LP and whether there is an “imitation” or “difference” competitive strategy in neighboring areas. *α*_3_ and *β*_3_ can identify the promotion or restriction of ER and LP. Therefore, the combination of the two aspects can reflect the form of interaction between ER and LP (Table 1).

*W_ij_* is the spatial weight matrix, which contains the geographic location relationship between city *i* and city *j*. In the context of local competition, local governments tend to imitate the behaviors of cities in geographical proximity or at a similar economic level. In this study, we used three spatial weights, namely a spatial proximity matrix (*W_G_*), a spatial distance matrix (*W_D_*), and an economic distance spatial weights matrix (*W_ED_*), to ensure the robustness of the estimation results. The spatial proximity matrix is a non-stochastic, predetermined, and contiguity-based binary matrix in which each element *W_ij_* is set to be 1 if city *i* and city *j* share a common border, and 0 otherwise. The spatial distance matrix was constructed useing *W_ij_* = (1/*d_ij_*), where *d_ij_* represents the Euclidean distance between city *i* and city *j*.

Considering that the competition between regions is more intense when the regions have similar levels of economic development [23], we used a matrix in which the geographical distance and economic distance interact. First, we calculated the reciprocal of the distance between cities to measure the Euclidean distance (1/*d_ij_*). Secondly, the reciprocal of the difference in per capita GDP between the two cities was calculated to measure the economic distance (1/|*pgdp_i_* − *pgdp_j_*|). Then, the Euclidean distance and economic distance were multiplied to obtain the spatial economic distance weights matrix. The matrix *W* is commonly row-standardized such that the elements of each row sum to 1.

### 3.5. Control Variables

We included different sets of control variables for the two equations. In the ER equation, environmental decentralization (*ED*), economic development level (*pgdp*), wage level (*wage*), industrial structure (*Sec*), and unemployment rate (*unem*) were used as control variables. Environmental decentralization reflects the degree of the central government’s intervention into local environmental protection and the incentives for and constraints on local governments’ environmental governance [48]. The greater the decentralization, the higher the autonomy of the local environmental management authority and the fewer the environmental protection incentives and supervision mechanisms [49]. Due to the lack of data at the city level, and the allocation of environmental protection personnel at the prefecture level being consistent with that at the provincial level, we multiplied the location quotient of personnel in the environmental protection system by 1 − (*gdp_it_*/*gdp_t_*) to represent the environmental decentralization in year *t* of province *i* [11].

Per capita GDP and wage levels reflect the economic income situation of the region. The higher the income level, the stronger the investment in local pollution control and the higher the demand for environmental quality [13]. We measured the per capita GDP and the average wage at the city level. The industrial structure is expressed by the proportion of the secondary industry in the GDP. The higher the proportion of the secondary industry, the easier it is for economic development to be affected by local governments and the more inclined governments are to use ER as a policy tool for economic competition because industrial interest groups may resist heavy regulation and lobby against strict law enforcement measures. The unemployment rate is characterized by the proportion of registered unemployed persons in urban areas to all employed persons. The higher the unemployment rate, the greater the pressure on governments to passively implement ER, and the greater the motivation to protect and develop polluting enterprises with a large employment capacity [23].

In the LP equation, we used fiscal decentralization (*fiscal*), industrial structure (*Sec*), foreign investment level (*fdi*), proportion of state-owned enterprises (*Soe*), capital–labor ratio (*Zbld*), and economic growth rate (*GZ*) as control variables. The mismatch between the fiscal power and administrative power of local governments will increase the incentives that local governments have to protect pollution-intensive industries. The ratio of fiscal revenue to total fiscal expenditure was used to measure fiscal decentralization [17]. The areas where foreign investment is intensive are mainly economically developed areas, and the investment industries are mainly clean production industries. The high level of marketization and the industrial structure make cities less dependent on the taxation of pollution-intensive industries. FDI was measured by the proportion of GDP it represents. The industrial structure was measured in the same way as in the first equation. The competitiveness and trade of industrial products incentivize cities with high industrial proportions to protect local enterprises.

State-owned enterprises have the characteristics of a large scale, strong employment absorption, and prominent political connections, which provide local governments with greater incentives to protect state-owned enterprises [22]. We used the ratio of the number of state-owned enterprises in pollution-intensive industries to the total number of enterprises to measure the proportion of state-owned enterprises. Industrial firms with outdated technology are less competitive, and it is easy to trigger LP behaviors. The capital–labor ratio of pollution-intensive industries in each city was used to measure the regional technology level. Human capital was measured by the number of college students per 10,000 people. Human capital is related to a region’s industrial competitiveness and technological level, the improvement of which will reduce the motivation of local governments to protect pollution-intensive industries. It also reflects citizens’ environmental awareness [50]. The demand for local governments to pursue economic growth is the root cause of LP [14]. The city-level GDP growth rate was used to control for the demand for regional economic growth.

### 3.6. Data Sources and Processing

We used a panel dataset of 285 Chinese prefecture-level cities for the period 2003–2013. There are 297 prefectures in total according to the administrative divisions, excluding Hong Kong, Macao, and Taiwan. However, due to the serious lack of data on 12 prefectures, we used the remaining 285 prefectures for the empirical analysis. The reason for using this period is that the industry data and enterprise data at the prefecture level were mainly obtained from China’s Industrial Enterprise Database and economic census data, and only data until 2013 are available for both. Moreover, this research period covers the continuous improvement of China’s ER system and governance of LP behavior. The Chinese government has paid more and more attention to the construction of an ecological civilization and market system, which is important when analyzing the behavior dilemma and changes in the conflict between ER and LP between local governments.

The research data were gathered from China’s Industrial Enterprise Database (2003–2013), economic census data (2003, 2008, and 2013), the Chinese Environmental Statistical Yearbook (2004–2014), the Chinese Industrial Statistical Yearbook (2004–2014), and the Chinese City Statistical Yearbook (2004–2014). China’s Industrial Enterprise Database can be obtained from the Express Professional Superior (EPS) data platform, while the other Statistical Yearbooks can be obtained from the National Bureau of Statistics in China.

In view of the problems of different statistical calibers and abnormal indicators in China’s Industrial Enterprise Database, we eliminated abnormal industrial output values and taxation data. The two-digit industries belonging to Chinese Industrial Classification codes GB/4754-2002 and GB/4754-2011 in different years were uniformly matched to GB/4754-2011. The matching process unified the company’s identification information based on administrative region division codes, postal codes, and other identification information into 285 city units. When calculating the LP index, the enterprises that directly belonged to the central or provincial government were excluded. In order to eliminate interference due to inflation factors, taking 2003 as the base period, we used the output-price deflator to flatten the data on industrial output values. The GDP deflator was used to smooth the price indicators, such as the per capita GDP, FDI, and employee wages.

## 4. Spatial Patterns of ER and LP

### 4.1. Spatial Distribution Characteristics of ER and LP

**Figure 2:**
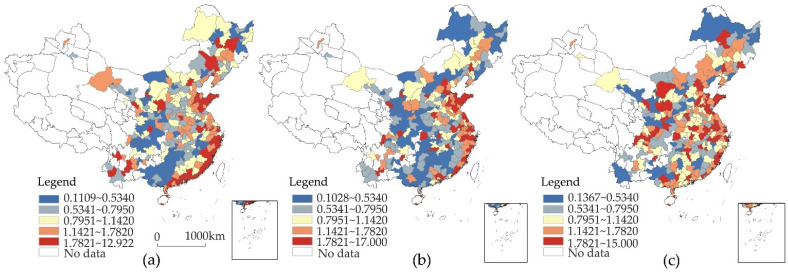
The spatial distribution and evolution of ER intensity. (**a**) The intensity of environmental regulation in 2003; (**b**) the intensity of environmental regulation in 2008; (**c**) the intensity of environmental regulation in 2013.

It can be seen from Figure 2 that the distribution of ER intensity is high in the east and low in the west, and the evolution of high-value areas shows a trend from contraction to expansion. Specifically, in 2003, areas with high ER intensity were mainly located in the coastal areas of the eastern region, while low-value areas covered the central and western regions. In 2008, the ER intensity in some cities in Guangdong, Fujian, and Zhejiang declined. The scope of the high-value areas decreased, and the scope of the low-value areas continued to expand. Rapid industrialization and urbanization led to the continuous deterioration of the environment, and it seemed as though ER had given in to economic growth. In 2013, with the continuous improvement of the environmental governance system, the overall ER intensity improved, and the high-value areas continued to expand to the central and western regions. Inland provincial capital cities and some cities in Sichuan, Guangxi, and Shaanxi entered the first tier, and the ER level in most of the remaining cities improved. The global spatial autocorrelation of the ER intensity confirms this transition, and its Moran’s *I* value is significantly positive and has a downward trend (Table 2). The agglomeration characteristics of high and low ER tend to evolve into spatially discrete distributions.

**Figure 3:**
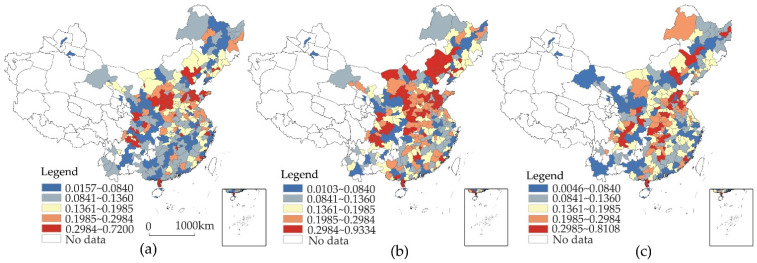
The spatial distribution and evolution of the LP index. (**a**) Local protection index in 2003; (**b**) local protection index in 2008; (**c**) local protection index in 2013.

The LP index has changed from high in the north and low in the south to high in the west and low in the east (Figure 3). The LP index not only reflects the degree of dependence of local and municipal tax sources on pollution-intensive industries, but also illustrates the transformation of the industrial structure of various cities. In 2003, the high-value areas were mainly concentrated in cities north of the Yangtze River. Most cities south of the Yangtze River had a low degree of tax reliance on pollution-intensive industries. In 2008, the LP index constituted a contiguous and concentrated high-value area in Shanxi, Henan, Shandong, Sichuan, Hebei, and Inner Mongolia, and most cities in Hubei, Hunan, Guangxi, Guangdong, and Fujian had an increased LP index. This is related to the spatial expansion of pollution-intensive industries [2,51] and is also a crucial factor in the overall reduction in the ER intensity at the same time. In 2013, the low-value areas of the LP index continued to expand, and the high-value areas were confined to cities such as Shandong, Henan, Sichuan, and Hubei. The Moran’s *I* value showed a downward trend, and the spatial distribution tended to be dispersed (Table 2). This phenomenon reflects the fact that the tax dependence of cities on pollution-intensive industries had decreased, and this may have been the result of industrial transformation and upgrading or enhanced ER. The result also shows a spatial correspondence between the expansion and contraction of ER and LP, which requires further analysis.

### 4.2. Spatial Correlation Analysis of ER and LP

We further calculated the ER spatial lag (*ER-GOV*) and LP spatial lag (*GOV-ER*) using Geoda and, in this section, we discuss the local spatial autocorrelation features of both spatial correlations. The results are shown in Table 2. The table shows that the ER intensity and the LP index generally exhibit a significant negative spatial correlation and spatial spillover effects. The correlation reflects the relationship between ER and LP to some extent. However, the degree of association presents an inverted U-shaped evolution trend and is no longer significant in 2012–2013, which is related to the transition of the two variables from agglomeration to a dispersed distribution.

**Figure 4:**
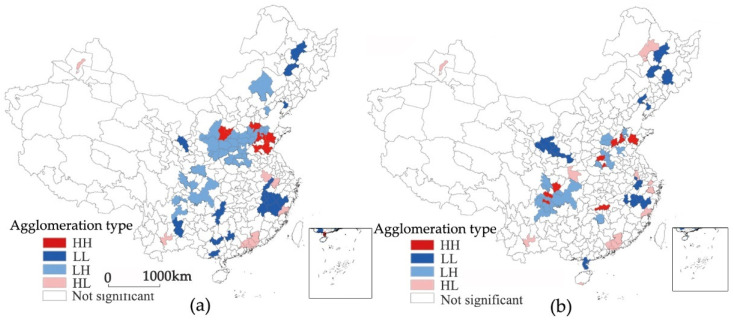
Bivariate LISA clustering of ER intensity. (**a**) Spatial lag of ER intensity in 2004; (**b**) spatial lag of ER intensity in 2011.

The bivariate local spatial autocorrelation can reflect the local spatial correlation of ER and LP. In this study, we selected 2004 and 2011 for LISA clustering. As shown in Figure 4, the inhibitory effect of ER on LP increases. In the bivariate local spatial autocorrelation of the lagging ER, the increase in the number of HL-type cities and the decrease in the number of LH-type cities reflect how the increase in the ER contributes to the transformation of the green industrial structure of cities. The increase in the number of HH-type cities also shows that the cities dependent on taxation sources of pollution-intensive industries have also paid more attention to environmental governance. Specifically, the HLtype is mainly concentrated in the Pearl River Delta region, and its policy focus on innovation and replacing obsolete industries makes it a model for the coordination of sustainable environmental and economic development. The contiguous areas of the LH type in Shaanxi, Shanxi, Henan, Hebei, and Shandong have contracted to eight cities, indicating that the region’s environmental governance and industrial structure transformation capabilities have improved. However, Sichuan has expanded from five cities to twelve cities, and the expansion of polluting industries may affect the improvement of ER. HH-type and LL-type cities have a relatively limited restraining effect on LP, which is reflected by the ER. However, the number of LL-type cities is constantly shrinking, and HH-type cities tend to be dispersed and to have partially transformed from the LH type (Zhengzhou, Nanchong, etc.), which to some extent reflects the improvement of the overall environmental governance capacity.

**Figure 5:**
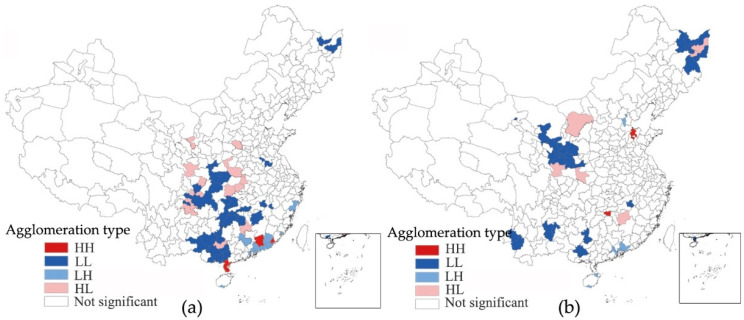
Bivariate LISA clustering of the LP index. (**a**) Spatial lag of the local protection index in 2004; (**b**) spatial lag of the local protection index in 2011.

Figure 5 shows the spatial relationship between LP and ER. Cities that have a significantly lagging LP bivariate spatial autocorrelation are mainly distributed in the south-central regions and northeastern regions, with the LL, LH, and HL types as the main types, and the spatial agglomeration distribution varies substantially. In 2004, the HL-type cities are mainly distributed in Henan, Hunan, and Sichuan, covering 15 cities. The dependence on tax sources of pollution-intensive industries may be an important reason to restrict the effectiveness of ER. However, in 2011, except for the fact that Ji’an and Shuangyahe changed from the LL type to the HL type and Ordos entered this type, the number of HL-type cities decreased to five, indicating that significant areas in which ER was restricted by LP had decreased. The LH type is mainly concentrated in the Pearl River Delta region, reflecting the significant environmental governance in this area and the weak incentives for local governments to protect pollution-intensive industries. The spatial distribution of the LL type changed from being concentrated in Hubei, Hunan, and Guangxi to spreading into the northwestern and northeastern regions. Since the dependence on pollution-intensive industries as a taxation source is relatively low, this reflects that low LP has a limited restraining effect on ER.

## 5. Empirical Results and Analysis

According to the theoretical analysis and spatial pattern analysis, there is a two-way causal relationship between ER and LP, and they have the characteristics of spatial agglomeration and spatial correlation. Therefore, the spatial simultaneous equations model constructed in this paper is reasonable. The generalized spatial three-stage least square (GS3SLS) estimation method proposed by Kelejian and Prucha [52] was used to estimate the equation using the fixed effect of the panel data, which can effectively solve the problems of the endogeneity of spatial simultaneous equations, cross-equation correlations, spatial endogeneity, and spatial correlations between error terms [47,53]. The instrumental variables in the model were the exogenous control variables and their spatial weighted variants [54]. The collinearity and correlation of variables were analyzed before the regression analysis, and the result shows that the VIF is less than 8 and the correlation coefficient is less than 0.5, indicating that no significant multicollinearity exists.

### 5.1. Analysis of Whole-Sample Estimation Results

The baseline regression result is shown in Table 3. Specifically, models (1), (3), and (5) are the estimated results of the LP equations, and models (2), (4), and (6) are the estimated results of the ER equations. All estimated results under the three spatial weight matrices based on *W_G_*, *W_D_*, and *W_ED_* are consistent, which demonstrates the robustness of the results.

In the LP equation, the spatial lag term of LP (W*Gov) and that of ER (W*ER) are significantly positive, indicating that LP incentives have regional “imitation behavior”. The economic competition between cities is an important reason for the existence of LP of pollution-intensive industries. When the neighboring areas strengthen the intensity of ER, it is a dominant strategy for local governments to increase the scale of polluting industries to increase the fiscal revenue and promote economic growth. In the ER equation, the coefficient of LP (*Gov*) is significantly negative, especially the coefficient in the model with the economic distance as the weight, which shows that LP behaviors for the purpose of economic growth and increasing fiscal and tax income will restrict the improvement of ER [8]. The spatial lag item of ER (W*ER) is significantly positive, indicating that there exists spatial correlation in the implementation of ER. Combined with the imitation behavior of ER by local governments and the restrictive effect of LP on ER, the results support the view that there is a “race to the bottom” nationwide in China. This is consistent with the research conclusions of Wu et al. [23].

However, the model proves that the restrictive effect of LP on ER is not one-way. The effect of ER on LP in the model is shown to be in the form of “imitation and restriction”, indicating that there is an interaction between the two, which is consistent with the aforementioned theoretical analysis. Combining the spatiotemporal pattern evolution of the two variables with the fact that the lag term of the LP (W*Gov) in the ER equation is a significantly positive value shows that the increase in the ER intensity is a gradual process carried out by the central government in order to strengthen the environmental management of local governments in response to environmental degradation caused by local government competition for polluting industries. It not only promotes the green transformation of local industries [55] but also weakens local incentives to protect pollution-intensive industries. The competition for ER between local governments is not only a “race to the bottom” but also a “race to the top” in the competition for cities’ interest and environmental governance.

In terms of control variables, the effects of environmental decentralization, per capita GDP, industrial structure, and unemployment rate on ER are all in line with expectations. The relationship between the wage level of employees and the ER, however, is not in line with expectations. This may have been caused by some cities (Baotou, Jiayuguan, etc.) in which the high income is closely related to pollution-intensive industries. The impact of foreign investment, industrial structure, fiscal decentralization, human capital, and GDP growth on LP is in line with expectations, indicating that economic growth and fiscal decentralization are important reasons for LP of pollution-intensive industries. The impact of the proportion of state-owned enterprises on LP is significantly negative. The LP index uses the tax proportion as a measurement, and the result reflects to a certain extent that the efficiency of state-owned enterprises is lower than that of other types of enterprises [37] and that the scale of private enterprises in China is increasing.

### 5.2. Analysis of Estimated Results by Period and by Region

The previous analysis showed the coexistence of a “race to the bottom” and a “race to the top” in a local space, so it is necessary to analyze whether there is temporal and spatial heterogeneity in the relationship between ER and LP. As the government determines the competition strategy, the interaction and transmission between cities with similar levels of economic development are more meaningful. Thus, only the economic distance spatial weight matrix (*W_ED_*) is used in this section. The results are shown in Table 4.

#### 5.2.1. Analysis of Estimated Results by Period

Taking 2008 as the split point, we divided the sample into two stages to analyze the temporal heterogeneity of the relationship between ER and LP. In 2008, the State Environmental Protection Administration was renamed to the Ministry of Environmental Protection. The central government promoted the substantial improvement of environmental governance capabilities and systems. In the same year, the anti-monopoly law was promulgated and implemented. The central government strengthened the supervision and vertical management of local governments, gradually reduced governmental intervention into the market, and weakened the LP in the field of environmental protection.

Comparing the estimated results for the period 2003–2007 and the period 2009–2013, the absolute values of the coefficient of both the LP index (*Gov*) in the ER equation and the spatial lag term of the LP index (W*Gov) in the LP equation have increased, indicating that the “imitation and restriction” effect of LP on ER has increased. This seems to contradict the central government’s weakening of LP. However, the mismatch between administrative power and fiscal power and the LP incentives of local governments to pursue economic growth have not disappeared, and the LP practices are becoming increasingly concealed [22]. The impact of the financial crisis on the manufacturing industries in various regions has also made local governments compete more intensely for pollution-intensive industries in order to ease the economic downturn and increase the momentum of economic development.

The role of ER in LP has changed from “imitation and invalidity” to “imitation and restriction”, indicating that there are thresholds on the restrictive effect of ER on LP. Facing pressure from the central government and the public, the refinement of the indicators for pollution restrictions, and the improvement of the environmental performance evaluation system, local governments’ ER behaviors tend to be better [10,51]. This restrains the blind expansion of local polluting industries due to the insufficient amount of green innovation and technology. This supports our theoretical analysis, that is, the improvement of environmental regulation helps to curb local protectionism, and the competition for environmental regulation changes from a “race to the bottom” to a “race to the top”.

In terms of other factors that affect the intensity of ER, environmental decentralization has become significantly negative, and wage levels have become significantly positive, indicating that the central government’s centralized management of environmental governance and the public’s demand for high-quality environments play an important role in increasing the intensity of ER [12,56]. The coefficient of the influence of the industrial structure and unemployment rate on the ER has changed from a significantly negative value to a non-significantly negative value, which may be related to the increasing emphasis on the growth of clean industries and increasing the industrial green technology level in local industrial development.

#### 5.2.2. Analysis of Estimated Results by Region

From a regional perspective, the interaction between ER and LP in the three major regions of the east, the central region, and the west is different (Table 4). According to the classification, eastern China includes 11 provinces: Beijing, Tianjin, Hebei, Liaoning, Shanghai, Jiangsu, Zhejiang, Fujian, Shandong, Guangdong, and Hainan. Central China includes eight provinces: Shanxi, Jilin, Heilongjiang, Anhui, Jiangxi, Henan, Hubei, and Hunan. Western China includes 12 provinces: Sichuan, Chongqing, Guizhou, Yunnan, Shaanxi, Gansu, Qinghai, Ningxia, Xinjiang, Inner Mongolia, Guangxi, and Tibet. In this study, cities were divided into three regions according to their provinces.

The impact of ER in the eastern region on LP is in the form of “imitation and restriction”, the impact of LP on ER is in the form of “imitation and invalidity”, and the competition for ER between cities is a “race to the top”. The reason for this is that the economic development level and environmental governance capabilities of the eastern region are relatively high. The enhancement of ER and the industrial transformation and upgrading policies forced pollution-intensive industries to transfer to underdeveloped regions [3,51], while the strong ability to obtain fiscal and tax revenue and the momentum of the diversified economic development weakened local government incentives to protect pollution-intensive industries.

The effect of ER in the central region on LP is in the form of “independence and restriction”, the effect of LP on ER is in the form of “imitation and restriction”, and the GOV coefficient in the ER equation is −3.64, which is much higher than that in the eastern and western regions. The central region is highly dependent on tax sources from pollution-intensive industries and is at the forefront of transferring enterprises from the eastern region [2]. The demand for the economy to catch up and economic growth has increased the motivation of local governments to protect and compete for tax sources of pollution-intensive industries. However, due to the weak innovation compensation effect of ER [42], the expansion of pollution-intensive industries affects the environmental governance. With strict ER and the need for industrial transformation and upgrading, the central region’s reliance on pollution-intensive industry tax sources has decreased, but there is a large gap in the level of economic development and environmental governance capabilities within the region, and the competition in ER between cities has been different and strategy-oriented.

The effect of ER in the western region on LP is in the form of “independence and invalidity”, and the effect of LP on ER is in the form of “imitation and restriction”. The level of economic development in the western region is relatively lower. The heavy-industry-based economic system has strengthened the motivation of local governments to protect and compete for pollution-intensive industries while restricting the improvement of ER [23]. Due to the low level of environmental regulation in the western region, the effect of ER on LP is not significant, and there is no effective spatial spillover effect of ER between regions. To some extent, these results confirm that local administrations in the less-developed inland regions of China, which have relatively lower levels of development, tend to hold stronger pro-growth values and weaker pro-environment values [7].

In terms of control variables, the impact of *ED* and *sec* in the eastern region on ER is significantly negative, which is different from that the central and western regions. This shows that the transformation and upgrading of the industrial structure in the eastern region are mainly based on clean industries. The influence of the proportion of state-owned enterprises on LP in the eastern and western regions is 0.161 and −0.082, respectively, indicating that the production efficiency of state-owned enterprises in the eastern region is higher than that in the western region and that the profitability of these enterprises is higher. 

### 5.3. Robustness Test

To further verify the reliability of our results, we substituted the indicators of the core variable to test the robustness of the whole-sample estimation results (Table 5), which confirms that the empirical results presented in Table 3 are robust. First, we used Wu et al.’s ER intensity measurement method [23] as an alternative index, which is widely used but does not consider the differences in the industrial structure, to re-estimate Equation (8). Except for the fact that the impact coefficient of *lnsec* on ER is not significant, the estimation results are consistent with those given in Table 3. Second, we included the mining industry as a pollution-intensive industry and recalculated the LP index. The estimation results remained consistent with those given in Table 3. Third, we replaced the spatial weight matrix of *W_ED_* by *W_G_* and re-estimated Equation (8) with alternative indicators of ER and LP simultaneously. In the whole-sample estimates, the impact of ER on LP was not significant, but the value became significant when using the samples from 2009 to 2013.

## 6. Conclusions and Policy Implications

China’s economy has achieved an unprecedented amount of growth in the past 40 years, but it has also paid a heavy environmental price. The question of how to balance economic development and environmental protection is an important topic for promoting green development and an ecological civilization. Due to the trade-off between environmental protection and economic growth, local governments in China face a conflict between ER and LP, which has a complex impact on the development and location of pollution-intensive industries and enterprises. Although many scholars have investigated the ER and LP implemented by Chinese local governments, we know little about their interplay when dealing with pollution-intensive industries and enterprises. This study contributes to this field by constructing an analytical framework that integrates the three actors of the central government, the local government, and the polluting firm as well as their complex game, competition, and asylum relations and by looking at the relationship and interplay between ER and LP at the Chinese city level.

Our study shows that there were obvious temporal and spatial differences in the ER intensity, LP index, and spatial correlation of Chinese cities and confirms that there was an interactive relationship between ER and LP. The average ER intensity first decreased and then increased, the spatial distribution was high in the east and low in the west, and the high-ER-value areas expanded to inland areas, indicating that China’s environmental governance ability had been enhanced. The average LP index in Chinese cities first increased and then decreased, and the spatial distribution changed from high in the north and low in the south to high in the west and low in the east, indicating that the overall degree to which China protects polluting industries had decreased. ER and LP have spatial spillover effects, and there is a negative spatial correlation effect between them that has an inverted U-shape. LP has an inhibitory effect on the improvement of ER intensity, and the improvement of ER intensity was conducive to the weakening of LP behavior. Our study not only confirms the conclusion of Wu et al. [26] that there is “race to the bottom” competition in ER among Chinese local governments, but also finds evidence of “race to the top” competition in the eastern region with the improvement of regional environmental governance capabilities.

Our study also shows that there is temporal and spatial heterogeneity in the interaction between ER and LP. LP of pollution-intensive industries is a dominant economic strategy for local governments that inhibits increases in ER intensity. The restrictive effect of ER on LP has a certain threshold and the effect increased over time. With the increase in environmental governance from the central government, the restrictive effect of ER on LP began to be more obvious, and the “race to the bottom” among local governments in competition for ER changed to a “race to the top”. The strengthening of ER has significantly weakened the local incentives to protect pollution-intensive industries since 2008. However, due to the impact of the financial crisis in 2008, the competition of local governments for tax sources of polluting industries increased, which still restricted the improvement of the ER intensity. The interplay between ER and LP is different in the three regions. The ER strategy between cities in the eastern region is a “race to the top”, which has a stronger weakening effect on LP. In the central and western regions, local governments have greater motivation to compete for and protect the tax sources of polluting industries, and the inhibiting effect of LP on the improvement of the ER intensity is more prominent.

From our results, we can derive some policy implications. First, the motivation of local governments to compete for and protect the tax sources of polluting industries could weaken the implementation of ER. The central government needs to reform the fiscal and tax system and the government performance evaluation system to eliminate the root causes of local protectionism and vicious competition and strengthen the supervision of local government behavior. Second, strengthening ER will help to weaken the motivation of local governments to protect the tax sources of polluting industries. The central government should reform the environmental management system, the ecological environment performance evaluation system, and ecological compensation and payment transfer mechanisms, improve the top-down environmental governance system, and mobilize the enthusiasm of local governments. Local governments should coordinate the industrial system, technology, and market construction in the field of environmental governance and promote the construction of a green manufacturing system. Third, there is spatial heterogeneity in the interaction between ER and LP. The eastern region should continue to play a leading role in environmental governance and industrial transformation and explore a win–win path to economic growth and environmental protection. The central and western regions should formulate flexible environmental regulations, strictly enforce the access system for and constraints on polluting enterprises, develop a green and low-carbon circular production mode, and continuously improve their ecological and environmental governance capabilities.

This paper provides a new research perspective for understanding the behavior dilemma that local governments face when attempting to balance economic development and environmental protection. The findings of this paper may also provide a reference for China’s environmental policy research. However, because of the data limitations at the prefecture level in China, we were unable to analyze the differences in the impact of different ER policy types on LP and the strategic interaction between prefectures and cities. In addition, more diverse measurement data on ER and LP would be required to further verify the conclusions of this study. Since the ER and LP behaviors of local governments are influenced not only by each other, but also by urban heterogeneities, such as the attitude of city mayors [23], environmental decentralization [11], and public participation in the agglomeration economy [13], studies on the mechanism of interaction between ER and LP need to incorporate more influencing factors. There are great differences in local government behaviors among regions and provinces and within provinces. It would also be necessary to build a spatiotemporal panel weight matrix that can reflect the differences in geographical locations. These limitations provide opportunities for future research.

## Figures and Tables

**Table 1:** The functional form of ER and LP.

	*β*_3_ or *α*_3_ > 0	*β*_3_ or *α*_3_ < 0	*α*_3_ or *β*_3_ Not Significant
*α*_1_ or *β*_1_ > 0	Imitation and promotion	Imitation and restriction	Imitation and invalidity
*α*_1_ or *β*_1_ < 0	Difference and promotion	Difference and restriction	Difference and invalidity
*α*_1_ or *β*_1_ not significant	Independence and promotion	Independence and restriction	Independence and invalidity

**Table 2:** Univariate and bivariate global Moran’s *I* statistics for ER and LP.

Year	*E* *R*		*Gov*		*E* *R* *-* *Gov*		*Gov-* *ER*	
	Moran’s *I*	*p*-Value	Moran’s *I*	*p*-Value	Moran’s *I*	*p*-Value	Moran’s *I*	*p*-Value
2003	0.432	0.001	0.191	0.001	−0.065	0.007	−0.028	0.125
2004	0.328	0.001	0.229	0.001	−0.067	0.004	−0.043	0.069
2005	0.429	0.001	0.241	0.001	−0.091	0.001	−0.068	0.011
2006	0.429	0.001	0.198	0.001	−0.092	0.001	−0.065	0.032
2007	0.383	0.001	0.251	0.001	−0.068	0.005	−0.047	0.043
2008	0.442	0.001	0.258	0.001	−0.069	0.005	−0.047	0.034
2009	0.265	0.001	0.231	0.001	−0.056	0.012	−0.037	0.091
2010	0.270	0.001	0.260	0.001	−0.055	0.010	−0.050	0.027
2011	0.125	0.008	0.251	0.001	−0.025	0.045	−0.035	0.082
2012	0.098	0.016	0.248	0.001	0.004	0.427	−0.010	0.398
2013	0.065	0.040	0.236	0.001	0.004	0.368	−0.012	0.391

**Table 3:** Whole-sample estimation results under three spatial weight matrices.

Variable	*W_G_*		*W_D_*		*W_ED_*	
	(1)	(2)	(3)	(4)	(5)	(6)
	*Gov*	*ER*	*Gov*	*ER*	*Gov*	*ER*
W*ER	0.0359 ***	0.6326 ***	0.0127 *	2.4576 ***	0.0338 ***	1.0030 ***
W*Gov	0.6124 ***	3.9405 ***	1.4011 ***	8.0671 ***	0.4143 ***	4.9218 ***
*ER*	−0.0163 ***		−0.0108 *		−0.0186 ***	
*Gov*		−5.1052 ***		−4.8659 ***		−5.3739 ***
*ED*		−0.1572 **		−0.0592		−0.0745 *
*lnpgdp*		0.5398 ***		0.6633 ***		0.4081 ***
*unem*		−5.2749 ***		−7.3899 ***		−5.2810 ***
*lnwage*		−0.4507 ***		−0.4955 ***		−0.3973 ***
*lnSec*	0.1363 ***	−0.2729 **	0.1798 ***	−0.5160 ***	0.1379 ***	−0.5128 ***
*fdi*	−0.9305 ***		−0.6813 ***		−0.7485 ***	
*fiscal*	−0.1167 ***		−0.1463 ***		−0.1156 ***	
*Soe*	−0.0738 ***		−0.0633 ***		−0.0808 ***	
*GZ*	0.0026 ***		0.0017 **		0.0030 ***	
*lnhum*	−0.0074 ***		−0.0083 ***		−0.0057 ***	
*lnZbld*	−0.0202 ***		−0.0344 ***		−0.0259 ***	
*_cons*	−0.2813 ***	1.3410 **	−0.4952 ***	3.6943 ***	−0.2299 ***	2.3793 ***
*N*	3135					
*R* ^2^	0.2268	0.1270	0.2778	0.0578	0.2172	0.0532
*System R* ^2^	0.5673		0.2723		0.5190	

Note: ***, **, and * indicate significance at the 1%, 5%, and 10% levels, respectively.

**Table 4:** Estimated results by stage and by region.

GOV Equation	2003–2007	2009–2013	Eastern Region	Central Region	Western Region
W*Gov	0.4274 ***	0.5173 ***	0.1995 ***	0.4769 ***	0.4429 ***
W*ER	0.0078	0.0829 ***	−0.0288 ***	−0.0510 **	−0.0206
*ER*	−0.0058	−0.0642 ***	−0.0128 ***	−0.0507 **	0.0192
*lnSec*	0.1167 ***	0.0923 ***	0.3304 ***	0.2007 ***	0.1130 ***
*fdi*	−0.6116 ***	−1.0838 ***	−0.8820 ***	−0.4385 **	−1.2533 ***
*fiscal*	−0.1215 ***	−0.0718 ***	−0.1349 ***	−0.0997 ***	−0.1887 ***
*Soe*	−0.1622 ***	−0.0357 *	0.1606 ***	−0.0236	−0.0818 ***
*GZ*	0.0047 ***	0.0022 **	0.0076 ***	0.0009	0.0066 ***
*lnhum*	−0.0057 *	−0.0068 *	−0.0009	−0.0162 ***	−0.0029 *
*lnZbld*	−0.0249 ***	−0.0286 ***	−0.0239 ***	−0.0113 **	−0.0247 ***
*_cons*	−0.1584 ***	−0.3021 ***	−0.9731 ***	−0.4023 ***	−0.1630 **
**ER Equation**	**2003–2007**	**2009–2013**	**Eastern Region**	**Central Region**	**Western Region**
W*ER	0.8531 ***	0.8997 ***	0.9147 ***	0.1518	−0.1212
W*Gov	3.8463 ***	4.0965 ***	0.2904	1.2298 ***	0.9641 **
*Gov*	−4.3295 ***	−5.5762 ***	−0.4321	−3.6399 ***	−1.0375 ***
*lnpgdp*	0.6819 ***	0.2073 ***	0.9250 ***	0.1138 ***	0.2268 ***
*ED*	−0.0695	−0.1693 *	−0.7545 **	0.0517	−0.0490
*unem*	−8.3287 ***	−2.3046	−11.5641 ***	−2.8971 ***	−2.7032 *
*lnwage*	−0.6254 ***	0.1136 *	−0.9006 ***	−0.0897 *	−0.1201
*lnSec*	−0.8422 ***	−0.2183	−5.2688 ***	0.6825 ***	0.3142 ***
*_cons*	3.5607 ***	−3.4656 **	2.7116 ***	−1.4988 ***	−1.0036 *
*N*	1425	1425	957	1254	924
*Gov R* ^2^	0.2431	0.4235	0.3016	0.1761	0.2819
*ER R* ^2^	0.2716	0.3017	0.3153	0.3714	0.2185
System *R*^2^	0.4327	0.8012	0.5248	0.6276	0.3045

Note: ***, **, and * indicate significance at the 1%, 5%, and 10% levels, respectively.

**Table 5:** Results of the robustness test of adjusted variables.

Variable	Adjusted ER		Adjusted LP		Adjusted ER and LP (Full Sample)		Adjusted ER and LP (2009–2013)	
	*W_ED_*		*W_ED_*		*W_G_*		*W_G_*	
	(1)	(2)	(3)	(4)	(5)	(6)	(7)	(8)
	*ER*	*Gov*	*ER*	*Gov*	*ER*	*Gov*	*ER*	*Gov*
W*ER	0.7797 ***	0.0167 ***	1.0100 ***	0.0401 ***	0.5994 ***	0.0105 ***	0.6170 ***	0.0316 ***
W*Gov	2.3826 **	0.3408 ***	6.0779 ***	0.5606 ***	4.1521 ***	0.6878 ***	5.9831 ***	0.6588 ***
*ER*		−0.0085 ***		−0.2161 *		0.0022		−0.0180 ***
*GOV*	−5.105 ***		−6.5155 ***		−5.0425 ***		−6.2462 ***	
*ED*	−0.5457 **		−0.0968		−0.6190 ***		−0.5797 ***	
*lnpgdp*	1.0867 ***		0.3993 ***		1.4786 ***		0.7713 ***	
*unem*	−12.0229 ***		−5.2020 ***		−12.2835 ***		−6.3331 **	
*lnwage*	−1.1864 ***		−0.4035 ***		−1.2533 ***		0.0399	
*lnSec*	0.2387	0.1575 ***	−0.4602 ***	0.1088 ***	−0.0365	0.1217 ***	0.0745	0.1329 ***
*fdi*		−0.8525 ***		−0.7135 ***		−0.9869 ***		−1.0692 ***
*fiscal*		−0.1285 ***		−0.0763 ***		−0.0999 ***		−0.0363
*Soe*		−0.0826 ***		−0.0614 ***		−0.0523 ***		−0.0082
*GZ*		0.0031 ***		0.0025 **		0.0023 ***		0.0017
*lnhum*		−0.0065 ***		−0.0051 **		−0.0089 ***		−0.0108 ***
*lnZbld*		−0.0270 ***		−0.0214 ***		−0.0179 ***		−0.2882 ***
*_cons*	2.6516 **	−0.2729 ***	2.3213 ***	−0.2052 ***	0.4399	−0.2639 ***	−6.8736	−0.2882
*R* ^2^	0.2896	0.2571	0.0375	0.161	0.309	0.2755	0.1776	0.1784
*System R* ^2^	0.5614		0.2724		0.5136		0.6123	

Note: ***, **, and * indicate significance at the 1%, 5%, and 10% levels, respectively.

## Data Availability

The data used in this study can be obtained from the National Bureau of Statistics in China and the Express Professional Superior (EPS) data platform.
